# *Plin4* modulates lipid droplet accumulation and ferroptosis in neurons exposed to benzo[a]pyrene

**DOI:** 10.1038/s41420-025-02747-8

**Published:** 2025-10-06

**Authors:** Hongyu Sun, Zhirui Ma, Xingdi Guo, Jie Chen, Hui He, Xiaomin Tong, Tingyu Ji, Shihan Ding, Chaoli Zhou, Yi Lyu, Fengjie Tian, Jinping Zheng

**Affiliations:** 1 Department of Toxicology, School of Public Health, Shanxi Medical University, Taiyuan, China; 2 Shanxi Key Laboratory of Aging Mechanism Research and Translational Applications; Center of Healthy Aging; School of Public Health and Preventive Medicine, Changzhi Medical College, Changzhi, China

**Keywords:** Predictive markers, Neuroscience

## Abstract

Benzo[a]pyrene (B[a]P), an environmental neurotoxin, induces cognitive decline through ferroptosis-mediated mechanisms. Transcriptomic analysis (GSE75206) of B[a]P-exposed mouse hippocampus identified 1668 differentially expressed genes, with *Plin4* emerging as a key regulator linked to ferroptosis and lipid droplet (LD) accumulation. Behavioral tests confirmed hippocampal-dependent cognitive impairment and *Plin4* upregulation. Molecular analyses demonstrated ferroptosis activation, evidenced by altered expression of related genes (*Gpx4*, *Slc7a11*, *Ptgs2*) and biochemical markers of lipid peroxidation and iron imbalance. In HT22 cells, Benzopyrene-7,8-Diol-9,10-Epoxide (BPDE) dose-dependently elevated *Plin4* expression, inducing mitochondrial damage and ferroptosis. Silencing *Plin4* reversed BPDE-induced ferroptosis by restoring redox balance, reducing LD accumulation, and improving mitochondrial integrity. Mechanistically, *Plin4* amplifies B[a]P neurotoxicity by exacerbating iron overload and LD accumulation, sensitizing neurons to ferroptosis. This study identifies *Plin4* as a central mediator of environmental pollutant-induced neurodegeneration and proposes it as a therapeutic target for ferroptosis-related cognitive disorders.

## Introduction

Benzo[a]pyrene (B[a]P), a potent polycyclic aromatic hydrocarbon and pervasive environmental contaminant, is primarily introduced into the human body through inhalation of polluted air, cigarette smoke, and the consumption of charred foods [1, 2]. While B[a]P is well established as a carcinogen, accumulating evidence suggests it also exerts substantial neurotoxic effects. Both epidemiological and experimental studies have linked B[a]P exposure to cognitive decline and an increased risk of neurodegenerative disorders, including Alzheimer’s disease (AD), Parkinson’s disease, Huntington’s disease, and amyotrophic lateral sclerosis [3, 4]. These conditions are commonly associated with oxidative stress, dysregulated iron metabolism, and cellular dysfunction [5–7]. However, the molecular mechanisms by which B[a]P contributes to neurotoxicity remain incompletely understood.

Ferroptosis, a recently identified form of programmed cell death, has emerged as a critical mechanism in a range of pathological conditions, including neurodegeneration, cancer, and organ injury [8]. It is characterized by iron-dependent lipid peroxidation, leading to excessive generation of reactive oxygen species (ROS), oxidative membrane damage, and ultimately, cell death [9]. Unlike apoptosis or necrosis, ferroptosis is governed by a complex interplay between iron metabolism and oxidative stress [10, 11]. Given the essential role of iron in maintaining neuronal function, dysregulation of iron homeostasis has been increasingly recognized as a major contributor to neurodegenerative diseases [12, 13].

Lipid peroxidation is a central feature of ferroptosis, primarily resulting from the oxidation of polyunsaturated fatty acids, such as arachidonic acid and linoleic acid, which are highly vulnerable to reactive oxygen species (ROS) during oxidative stress [11]. Lipid droplets (LDs), which govern intracellular lipid storage, have been increasingly linked to the modulation of oxidative stress responses and cellular susceptibility to ferroptosis [14, 15]. Nevertheless, the precise role of LD-associated mechanisms in B[a]P-induced ferroptosis remains poorly understood.

Our prior investigations revealed that 7,8-dihydroxy-9,10-epoxybenzo[a]pyrene (BPDE), the biologically active metabolite of B[a]P, induces ferroptosis in both rat primary cortical neurons and human SH-SY5Y neuroblastoma cells in a dose- and time-dependent manner. These findings implicate ferroptosis as a central mechanism in B[a]P-induced neurotoxicity. Nevertheless, the precise molecular pathways by which B[a]P initiates ferroptosis—particularly those involving lipid droplet–associated processes—remain largely unexplored.

Therefore, this study aims to elucidate the molecular mechanisms underlying B[a]P-induced ferroptosis. By identifying key regulatory pathways and mediators, we seek to advance understanding of the neurotoxic effects of environmental pollutants and inform the development of therapeutic strategies to mitigate ferroptosis-related neurodegeneration.

## Results

### Identification of Key Gene *Plin4* in the hippocampus of B[a]P-exposed mice based on GEO data and bioinformatics analysis

In a prior study by Chepelev et al., behavioral assessments indicated impairments in learning and memory, suggesting significant neurotoxicity due to B[a]P exposure [16]. In their 2016 work, an acute B[a]P exposure model was established using adult male Muta™ Mice. Animals received oral doses of B[a]P at 0, 1, 35, or 70 mg/kg body weight per day for three consecutive days. Transcriptomic analysis revealed alterations in synaptic function—such as upregulation of N-methyl-D-aspartate receptor subunits *Grina* and *Grin2a*—without notable activation of DNA damage response genes, despite the presence of B[a]P-DNA adducts. These findings suggest that B[a]P-induced neurotoxicity may arise primarily from disrupted synaptic signaling rather than direct genotoxic damage.

**Fig. 1:**
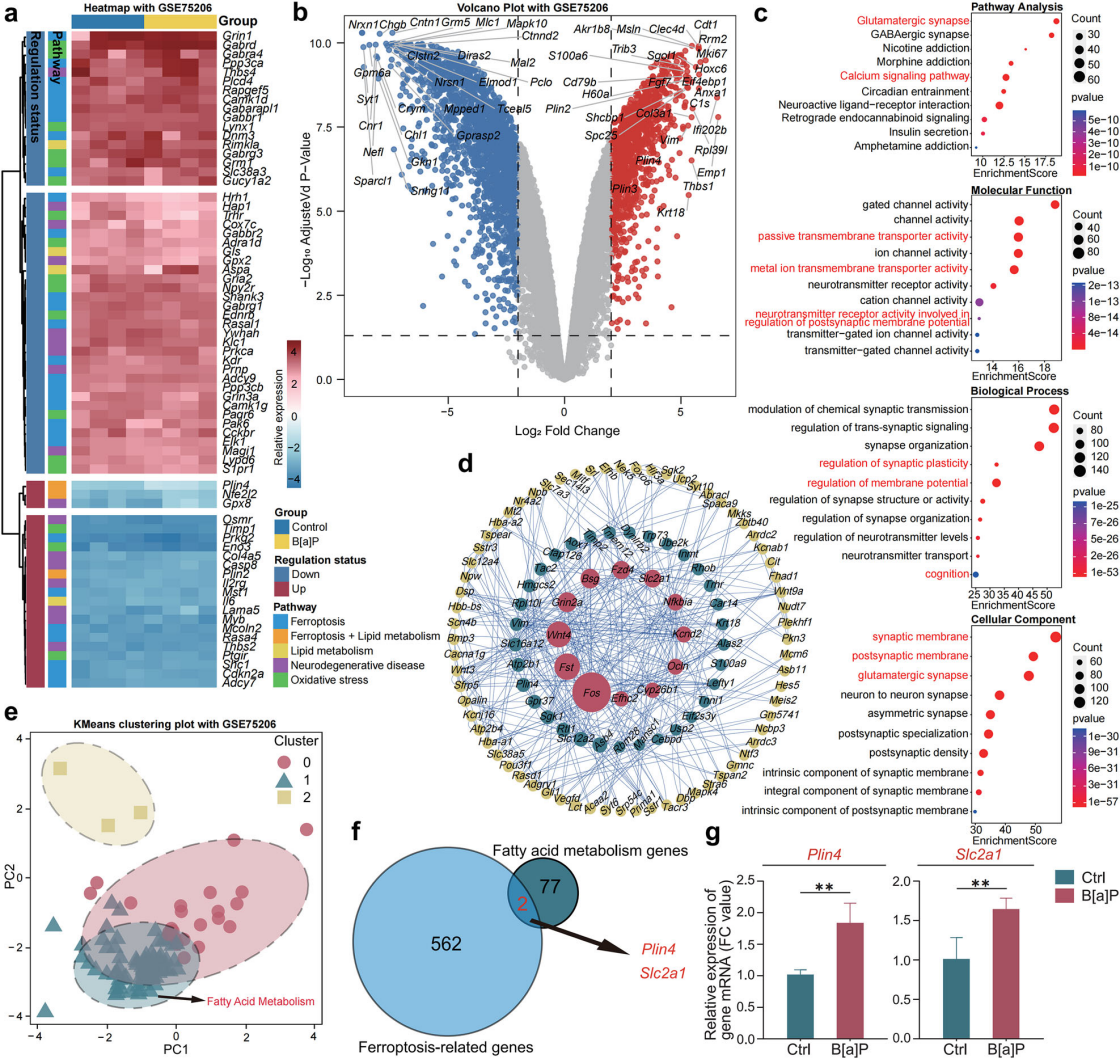
Bioinformatics Analysis Based on GEO Data Identifies Key Gene *Plin4* in Hippocampal Tissue of B[a]P-Treated Mice. **a** Heatmap showing gene expression changes in the hippocampus of mice following acute B[a]P exposure. **b** Volcano plot showing the overall distribution of differentially expressed genes, with upregulated and downregulated genes marked based on FDR correction (FDR *P*-value < 0.05, |log_2_FC| ≥ 3). **c** The GO and KEGG pathway enrichment analysis results show biological functions and signaling pathways associated with the DEGs, including neurotransmitter receptor activity, synaptic plasticity, membrane potential regulation, and neurodegenerative diseases such as AD and ferroptosis. **d** PPI network illustrating interactions between DEGs, with BC analysis identifying genes key to lipid metabolism and neurobiology. **e** KMeans clustering analysis categorizing genes into three clusters based on expression levels (log_2_FC) and BC scores, with Cluster 1 showing a strong association with lipid metabolism. **f** Venn diagram showing overlap between KMeans clustering and ferroptosis-related genes, identifying *Plin4* and *Slc2a1* as key genes, with *Plin4* significantly upregulated in hippocampal tissue of B[a]P-treated mice, suggesting its role in neurotoxicity. **g** Comparison of expression levels between *Plin4* and *Slc2a1*, with *Plin4* identified as a core gene in B[a]P-induced neurotoxicity. Data are presented as mean ± SEM(*n* = 4 mice per group) *P < 0.05, **P < 0.01, ***P < 0.001, ****P < 0.0001.

To further investigate the molecular underpinnings of B[a]P neurotoxicity, we conducted a comprehensive bioinformatics analysis using the hippocampal transcriptome dataset generated by Chepelev et al., available from the NCBI GEO database (accession number GSE75206). High-throughput RNA sequencing data from hippocampal tissues were analyzed to identify transcriptional changes associated with B[a]P exposure. Differential expression analysis (FDR-adjusted *P*-value < 0.05, |log₂FC| ≥ 3) revealed 1,668 significantly differentially expressed genes (DEGs). A heatmap illustrated the clustering patterns of gene expression profiles (Fig. 1a), and a volcano plot displayed the distribution of DEGs (Fig. 1b), highlighting the extent of transcriptional perturbation caused by B[a]P.

To elucidate the functional implications of these DEGs, we performed Gene Ontology (GO) and Kyoto Encyclopedia of Genes and Genomes (KEGG) pathway enrichment analyses (Fig. 1c). At the molecular function level, DEGs were significantly enriched in passive transmembrane transporter activity, metal ion transmembrane transporter activity, and neurotransmitter receptor activity—particularly in processes regulating postsynaptic membrane potential—suggesting critical roles in maintaining neuronal signaling and membrane stability. At the biological process level, enriched terms included synaptic plasticity, cognitive function, and membrane potential regulation. At the cellular component level, DEGs were mainly associated with synaptic and postsynaptic membranes as well as glutamatergic synapses. KEGG analysis further revealed enrichment in pathways related to neurodegenerative diseases, glutamatergic synapse signaling, calcium signaling, and notably, ferroptosis—implying a possible role for ferroptosis in B[a]P-induced neurotoxicity.

To examine gene–gene interactions and identify biologically relevant regulators, we constructed a PPI network using the STRING database (https://string-db.org/) and visualized it in Cytoscape. After removing non-interacting genes, we calculated betweenness centrality (BC) scores using the CytoNCA plugin to pinpoint hub genes with regulatory potential (Fig. 1d). This network analysis highlighted genes involved in adipocyte differentiation, fatty acid degradation, and lipid metabolism—processes increasingly recognized as critical in neural homeostasis and pathology.

We then normalized both log₂FC and BC scores and applied KMeans clustering to classify DEGs based on both expression magnitude and network centrality. This approach grouped genes into three distinct clusters (Cluster 0, Cluster 1, Cluster 2), with Cluster 1 showing the strongest association with lipid metabolic processes based on literature evidence (Fig. 1e). To further narrow down potential mechanistic targets, we intersected Cluster 1 genes with ferroptosis-related genes curated in the FerrDb database (http://www.zhounan.org/ferrdb), identifying two candidates: *Slc2a1* and *Plin4* (Fig. 1f). Comparative expression analysis demonstrated a more pronounced upregulation of *Plin4* in the hippocampus of B[a]P-exposed mice relative to *Slc2a1* (Fig. 1g). Given *Plin4*’s known role in lipid droplet formation and its emerging link to oxidative stress regulation, we identified *Plin4* as a potential key mediator of B[a]P neurotoxicity.

Taken together, our analysis suggests that B[a]P exposure leads to upregulation of *Plin4*, which may disrupt lipid metabolism homeostasis and promote ferroptosis—an iron-dependent form of programmed cell death characterized by lipid peroxidation. This mechanism could underlie the neuronal damage and cognitive deficits observed in B[a]P-exposed mice, as initially reported by Chepelev et al. We therefore propose that *Plin4*-mediated ferroptosis plays a critical role in B[a]P-induced neurotoxicity. This mechanistic insight not only advances our understanding of environmental pollutant-related neurodegeneration but may also offer a novel therapeutic target for mitigating B[a]P-associated cognitive impairment.

### Subacute Exposure to B[a]P Induces Ferroptosis and High Expression of *Plin4* in Mouse Hippocampal Tissue

**Fig. 2:**
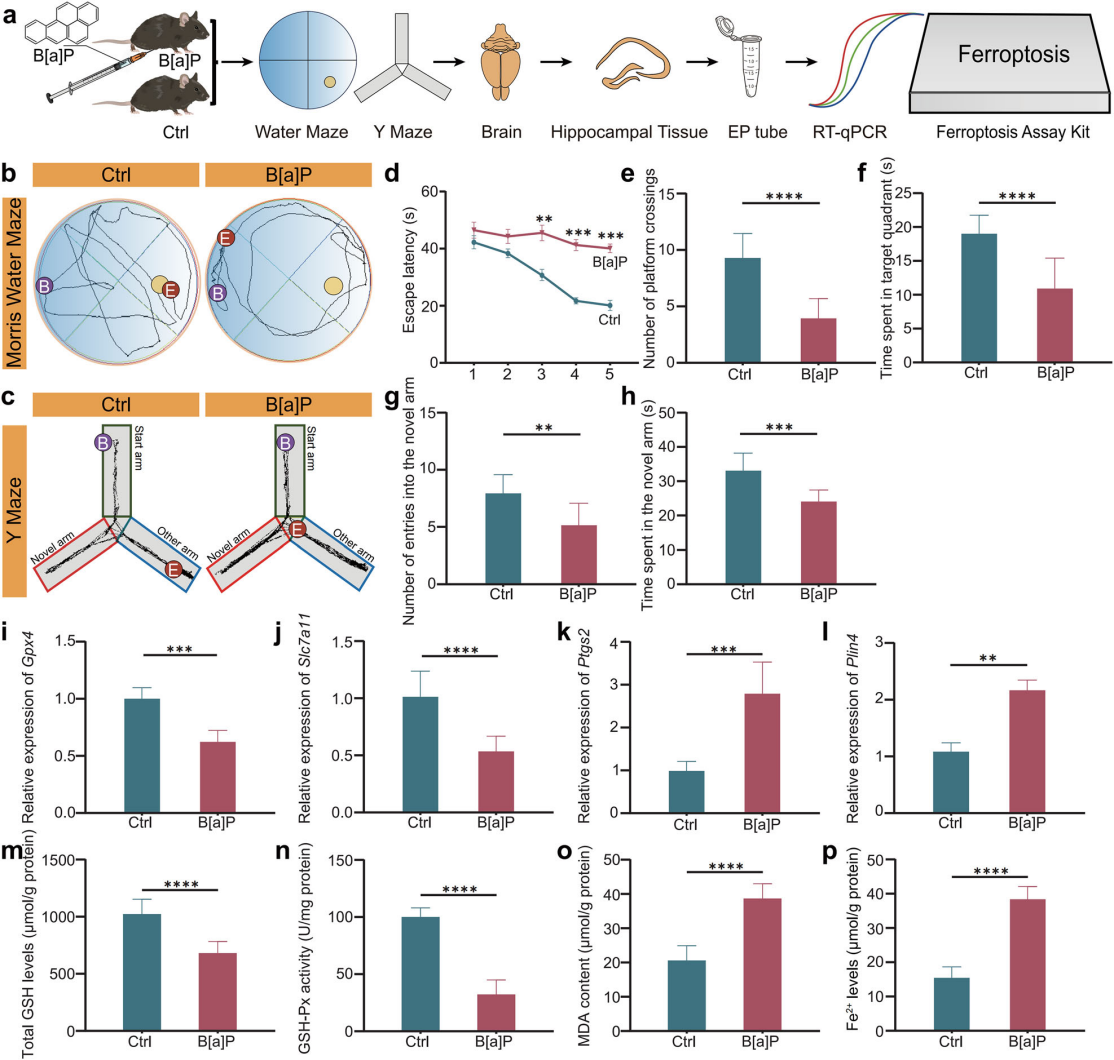
Subacute B[a]P exposure induces ferroptosis and upregulates *Plin4* expression in mouse hippocampal tissue. **a** Experimental workflow illustrating the steps involved in the construction of the B[a]P subacute exposure mouse model, followed by behavioral experiments and gene expression analysis. **b** Motion trajectory of mice in the MWM experiment. **c** Motion trajectory of mice in the Y-maze experiment. **d** Comparison of escape latency in the MWM experiment. **e** Comparison of platform crossings in the MWM experiment. **f** Comparison of time spent in the target quadrant in the MWM experiment. **g** Comparison of the number of new arm entries in the Y-maze experiment. **h** Comparison of the time spent exploring new arms in the Y-maze experiment. **i–l** qPCR results showing the relative expression levels of *Gpx4*, *Slc7a11*, *Ptgs2*, and *Plin4* in hippocampal tissue. **m** Total GSH levels in hippocampal tissue. **n** GSH-Px activity in hippocampal tissue. **o** MDA content in hippocampal tissue. **p** Fe²⁺ levels in hippocampal tissue. Data are presented as mean ± SEM(*n* = 6 mice per group, Ctrl and B[a]P groups) *P < 0.05, **P < 0.01, ***P < 0.001, ****P < 0.0001.

To test our hypothesis, we established a subacute B[a]P exposure mouse model to validate the bioinformatics analysis results. Mice received daily intraperitoneal injections of 10 mg/kg B[a]P for 30 consecutive days, successfully replicating subacute exposure conditions. Figure 2a outlines the experimental procedures, including model establishment, behavioral testing, and gene expression analysis. The movement trajectories of mice during the Morris water maze and Y-maze tests are shown in Fig. 2b, c, respectively.

In the water maze, B[a]P-treated mice exhibited significantly prolonged escape latencies (Fig. 2d), as well as fewer platform crossings and reduced time spent in the target quadrant (Fig. 2e, f). The Y-maze results further supported cognitive impairment, as treated mice showed decreased entries into and time spent in the novel arm (Fig. 2g, h), indicating impaired spatial memory and exploratory behavior.

To investigate the molecular mechanisms underlying these behavioral deficits, we conducted qPCR analysis of hippocampal tissues. Expression of *Gpx4* and *Slc7a11* was significantly downregulated (Fig. 2i, j), while *Ptgs2* expression was markedly upregulated (Fig. 2k) in the B[a]P-treated group. Notably, *Plin4* expression was significantly increased in the hippocampus, consistent with our bioinformatics predictions (Fig. 2l).

We further evaluated ferroptosis-related biochemical markers. GSH levels were decreased (Fig. 2m), whereas GSH-Px activity was significantly reduced (Fig. 2n). Additionally, MDA content and Fe²⁺ levels were both significantly increased (Fig. 2o, p), supporting the activation of ferroptosis.

Our bioinformatics analysis of the GSE75206 dataset identified *Plin4* as a candidate gene potentially involved in B[a]P-induced neurotoxicity. This was corroborated in vivo by our subacute exposure model, which demonstrated cognitive impairment alongside increased hippocampal *Plin4* expression. The observed molecular and biochemical changes—including reduced *Gpx4* and *Slc7a11*, elevated *Ptgs2*, and disrupted ferroptosis indicators—strongly implicate ferroptosis as a key mechanism in B[a]P-induced cognitive dysfunction. Together, these findings suggest that *Plin4* may contribute to neurotoxicity through modulation of ferroptosis, providing novel insights into the mechanisms of B[a]P-related brain injury.

### *Plin4* regulates ferroptosis in BPDE-induced hippocampal neurons

#### Upregulation of *Plin4* in BPDE-induced hippocampal neurons

**Fig. 3:**
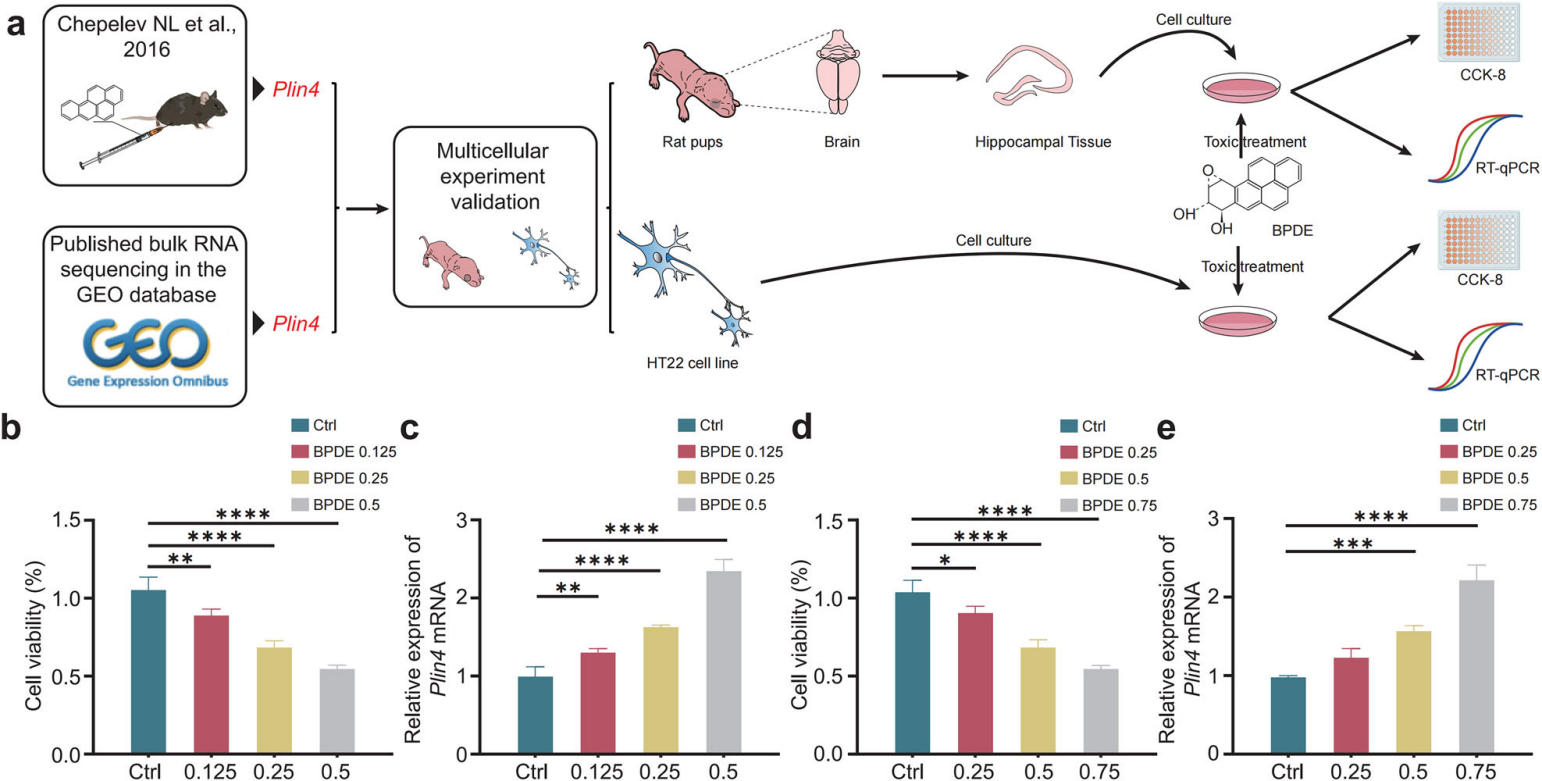
Expression of *Plin4* in B[a]P-induced hippocampal neuronal death. **a** Experimental workflow summarizing the steps of the current analysis. **b** Survival rate changes of primary mouse hippocampal neurons under different concentrations of BPDE treatment. The survival rate decreases as BPDE concentration increases. **c** Relative expression of *Plin4* in primary mouse hippocampal neurons under BPDE treatment, with a significant upregulation of *Plin4* expression. **d** Survival rate changes of HT22 cells under different concentrations of BPDE treatment, showing a dose-dependent decrease in survival rate as BPDE concentration increases. **e** Relative expression of *Plin4* in HT22 cells under BPDE treatment, with a significant upregulation of *Plin4* expression as BPDE concentration increases. Data are presented as mean ± SEM(*n* = 6 per group, BPDE concentrations in μM) *P < 0.05, **P < 0.01, ***P < 0.001, ****P < 0.0001.

Building on our in vivo observations that *Plin4* expression is upregulated in the hippocampus of B[a]P-exposed mice, we further explored the role of *Plin4* in B[a]P-induced neuronal injury at the cellular level. To dissect the specific contribution of *Plin4*, we conducted in vitro studies using primary mouse hippocampal neurons and the mouse hippocampal neuronal cell line HT22. Given that in vitro systems do not fully recapitulate the complexity of in vivo metabolic processes, we employed BPDE—the biologically active metabolite of B[a]P—as a surrogate for B[a]P exposure. The experimental workflow is depicted in Fig. 3a.

Cells were treated with increasing concentrations of BPDE (0 μM, 0.25 μM, 0.5 μM, 0.75 μM). As the concentration rose, both primary neurons and HT22 cells exhibited a dose-dependent decline in viability (Fig. 3b, d). Meanwhile, *Plin4* expression was significantly upregulated in both cell types (Fig. 3c, e).

Primary mouse hippocampal neurons offer a physiologically relevant model that more closely mirrors in vivo conditions, while HT22 cells provide greater experimental reproducibility and technical ease. Using both models enabled cross-validation of our results, enhancing the robustness and biological significance of our findings.

In summary, our results demonstrate that neuronal *Plin4* expression increases in a dose-dependent manner following BPDE exposure, supporting the hypothesis that *Plin4* plays a critical role in the neurotoxic effects associated with B[a]P.

#### Silencing *Plin4* attenuates BPDE-induced ferroptosis in HT22 cells

**Fig. 4:**
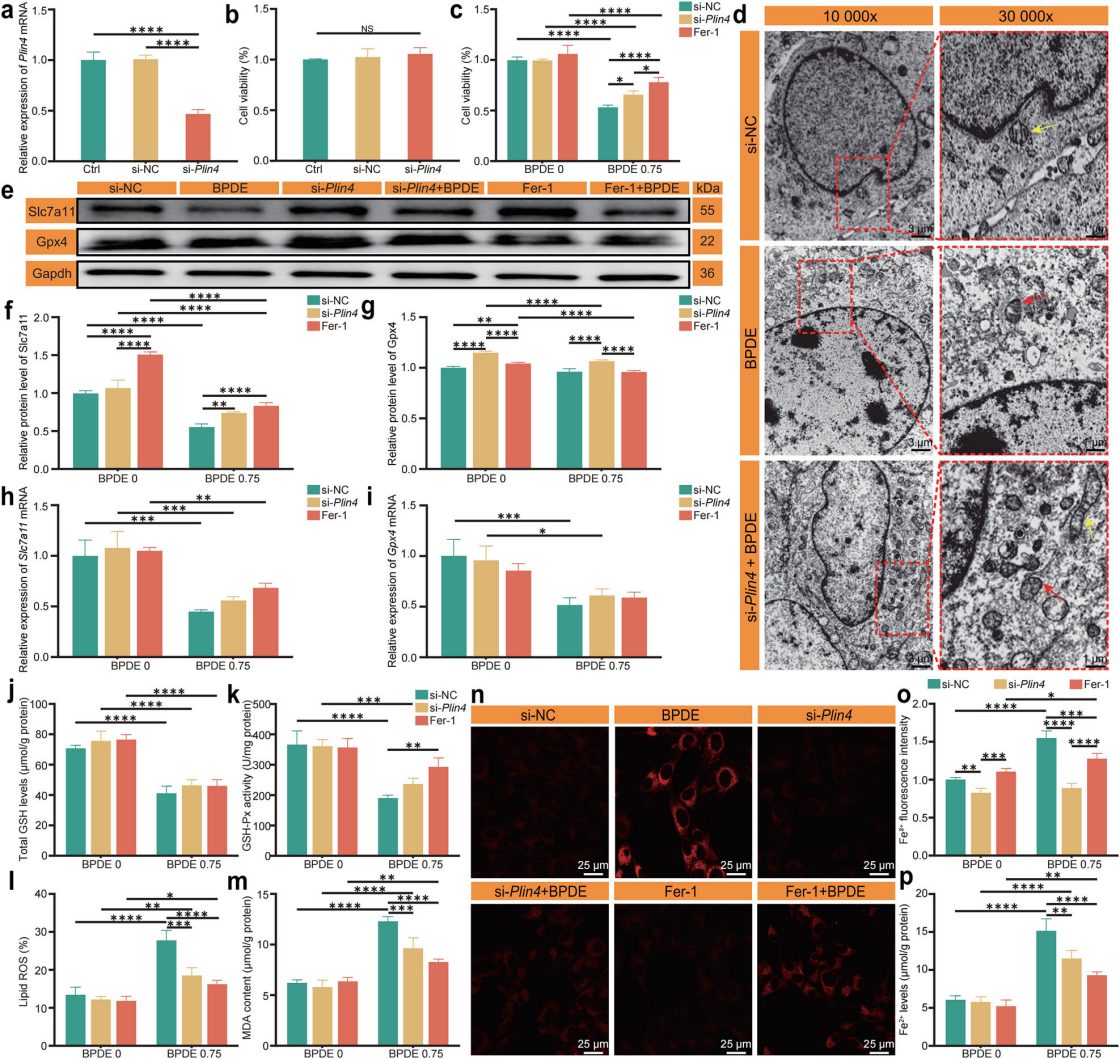
Role of *Plin4* in BPDE-induced ferroptosis in HT22 cells. **a** Schematic of the construction of the *Plin4*-silenced HT22 cell model. **b** Effect of *Plin4* silencing on HT22 cell survival rate. **c** Antagonistic effect of *Plin4* silencing on BPDE-induced HT22 cell ferroptosis, compared with the ferroptosis inhibitor Fer-1. **d** TEM images showing mitochondrial damage in HT22 cells after BPDE treatment. Yellow arrows indicate normal mitochondria, while red arrows indicate damaged mitochondria. **e** Protein bands of Slc7a11 and Gpx4, showing the expression changes after BPDE treatment and *Plin4* silencing. **f** Relative expression level of Slc7a11 protein. **g** Relative expression level of Gpx4 protein. **h** Relative expression level of *Slc7a11* mRNA. **i** Relative expression level of *Gpx4* mRNA. **j** Effect of BPDE treatment on total GSH content. **k** Effect of BPDE treatment on GSH-Px activity. **l** Effect of BPDE treatment on lipid ROS levels. **m** Effect of BPDE treatment on MDA content. **n** Confocal fluorescence images showing Fe²⁺ levels, illustrating the effect of BPDE treatment and *Plin4* silencing on Fe²⁺ levels. **o** Fluorescence intensity calculated using ImageJ software, showing the changes in Fe²⁺ levels in different groups. **p** Fe²⁺ level detected using a reagent kit, comparing Fe²⁺ levels between *Plin4* silencing and Fer-1 treatment groups. Data are presented as mean ± SEM (*n* = 6 per group). Group details: a-b: Ctrl, si-NC, si-*Plin4*. **c**, **f**–**m**, **o**–**p**: Two BPDE concentrations (0 μM and 0.75 μM), treated with si-NC, si-*Plin4*, or Fer-1. *P < 0.05, **P < 0.01, ***P < 0.001, ****P < 0.0001.

Based on our previous research, which showed a dose-dependent increase in *Plin4* expression in hippocampal neurons after BPDE exposure, we aimed to investigate the role of *Plin4* in BPDE-induced ferroptosis. Although bioinformatics analysis suggests a potential link between *Plin4* and ferroptosis, the precise mechanisms remain unclear. Our prior studies demonstrated that BPDE, a metabolite of B[a]P, induces ferroptosis in hippocampal neurons. However, how the lipid metabolism-related gene *Plin4* contributes to this process is still unknown. To explore the role of *Plin4* in BPDE-induced ferroptosis, we performed *Plin4* knockdown experiments and successfully established a *Plin4*-silenced HT22 cell model (Fig. 4a). The results in Fig. 4b showed that silencing *Plin4* did not significantly affect cell viability.

We then compared the effects of the ferroptosis inhibitor Ferrostatin-1 (Fer-1) and *Plin4* silencing on BPDE-induced ferroptosis in HT22 cells. BPDE treatment significantly reduced cell viability, which was partially restored by Fer-1. Similarly, *Plin4* silencing significantly improved cell viability after BPDE exposure, though the recovery was less than that observed with Fer-1. This suggests that *Plin4* plays a critical role in BPDE-induced ferroptosis, likely by regulating lipid peroxidation and iron metabolism (Fig. 4c).

Transmission electron microscopy (TEM) revealed that BPDE treatment caused mitochondrial cristae fragmentation, dissolution, and increased membrane density in HT22 cells. Notably, *Plin4* silencing partially alleviated these mitochondrial structural defects, suggesting a protective effect on mitochondrial integrity (Fig. 4d). Yellow arrows point to normal mitochondria, while red arrows highlight damaged ones. The restoration of mitochondrial morphology in the *Plin4*-silenced group suggests that *Plin4* may contribute to mitochondrial damage during ferroptosis, potentially through its regulation of lipid metabolism and oxidative stress.

To further elucidate the molecular mechanisms underlying BPDE-induced ferroptosis, we examined the expression of two key ferroptosis-related genes, *Slc7a11* and *Gpx4*, as well as their corresponding proteins, Slc7a11 and Gpx4. As shown in Fig. 4h, i, BPDE (0.75 μM) treatment led to a significant downregulation of *Slc7a11* and *Gpx4* mRNA expression levels, indicating transcriptional suppression of ferroptosis-inhibitory factors. At the protein level, Western blot analysis (Fig. 4e; full blot images are shown in Supplementary Material) and quantification (Fig. 4f, g) revealed a marked decrease in Slc7a11 and a moderate but statistically significant reduction in Gpx4 protein levels following BPDE exposure, supporting a role for BPDE in promoting ferroptosis.

Interestingly, knockdown of *Plin4* significantly restored Slc7a11 and Gpx4 protein expression (Fig. 4f, g), suggesting that *Plin4* contributes to BPDE-induced ferroptosis signaling. However, *Plin4* silencing had no statistically significant effect on the mRNA levels of *Slc7a11* and *Gpx4* (Fig. 4h and i), indicating that *Plin4* may regulate these proteins primarily at the post-transcriptional level, possibly through modulation of translation efficiency or protein stability. Notably, treatment with the ferroptosis inhibitor Fer-1 selectively reversed the BPDE-induced reduction in Slc7a11 protein levels, while Gpx4 protein expression remained largely unchanged, suggesting that BPDE-induced suppression of Gpx4 may occur through a distinct or less ferroptosis-sensitive mechanism. Together, these findings suggest that *Plin4* may facilitate BPDE-induced ferroptosis by promoting the post-transcriptional downregulation of Slc7a11 and Gpx4. Among them, Slc7a11 appears to be more tightly coupled to ferroptosis regulation. Thus, *Plin4* may represent a previously unrecognized modulator of ferroptosis under BPDE-induced stress conditions.

After BPDE exposure, GSH levels and GSH-Px activity were significantly reduced, while lipid ROS and MDA levels increased. However, silencing *Plin4* restored GSH levels and GSH-Px activity, while lipid peroxidation and MDA levels were significantly decreased, although not as strongly as with Fer-1 (Fig. 4j–m). These results suggest that silencing *Plin4* helps restore cellular antioxidant defenses, reducing lipid peroxidation and oxidative damage—key events in ferroptosis.

We also assessed Fe²⁺ levels using an iron ion assay kit and confocal microscopy (Fig. 4n). The results showed that BPDE treatment significantly increased Fe²⁺ levels, while both Fer-1 and *Plin4* silencing reduced Fe²⁺ levels, with the *Plin4*-silenced group showing effects similar to those of Fer-1 (Fig. 4o). Fluorescence intensity analysis revealed slightly lower Fe²⁺ levels in the *Plin4*-silenced group compared to the Fer-1 group (Fig. 4p). These findings reinforce the role of *Plin4* in regulating ferroptosis-related molecules and Fe²⁺ levels, suggesting that *Plin4* plays a role in maintaining iron homeostasis and preventing ferroptosis.

In conclusion, our study demonstrates that silencing *Plin4* protects HT22 cells from BPDE-induced ferroptosis. This protective effect likely involves the regulation of iron homeostasis, oxidative stress, antioxidant defenses, and mitochondrial function. By influencing lipid metabolism and mitochondrial integrity, *Plin4* emerges as a key regulator of ferroptosis and a potential therapeutic target for ferroptosis-related diseases. These findings offer new insights into lipid metabolism’s role in ferroptosis and suggest that targeting *Plin4* may provide novel strategies for treating neurodegenerative diseases linked to environmental toxicants.

#### *Plin4* may enhance the sensitivity of BPDE-induced ferroptosis in HT22 cells by regulating LD accumulation

To delve deeper into the mechanisms by which *Plin4* contributes to BPDE-induced ferroptosis in HT22 cells, we conducted a comprehensive literature review. Previous studies have shown that *Plin4*, a LD-associated protein, inhibits mitochondrial autophagy and promotes neuronal apoptosis, thereby facilitating the development and progression of neurodegenerative diseases. These findings imply a critical role for *Plin4* in cognitive dysfunction. Given the close interplay between LD metabolism, oxidative stress, and neuronal injury, we hypothesized that *Plin4* may regulate ferroptosis sensitivity by modulating aberrant LD accumulation under BPDE exposure.

LDs are essential organelles for neutral lipid storage and are involved in various physiological processes, including energy metabolism, steroid hormone synthesis, endoplasmic reticulum stress responses, mitochondrial function, and membrane maintenance. Recent studies suggest that beyond their metabolic functions, LDs may also contribute to the pathogenesis of neurodegenerative disorders. Under normal conditions, LDs are primarily distributed in glial cells, ependymal cells, and microglia within the nervous system [17, 18]. However, under pathological conditions such as neuronal aging or oxidative stress, LDs abnormally accumulate in neurons, a phenomenon closely linked to neurodegenerative disease progression.

**Fig. 5:**
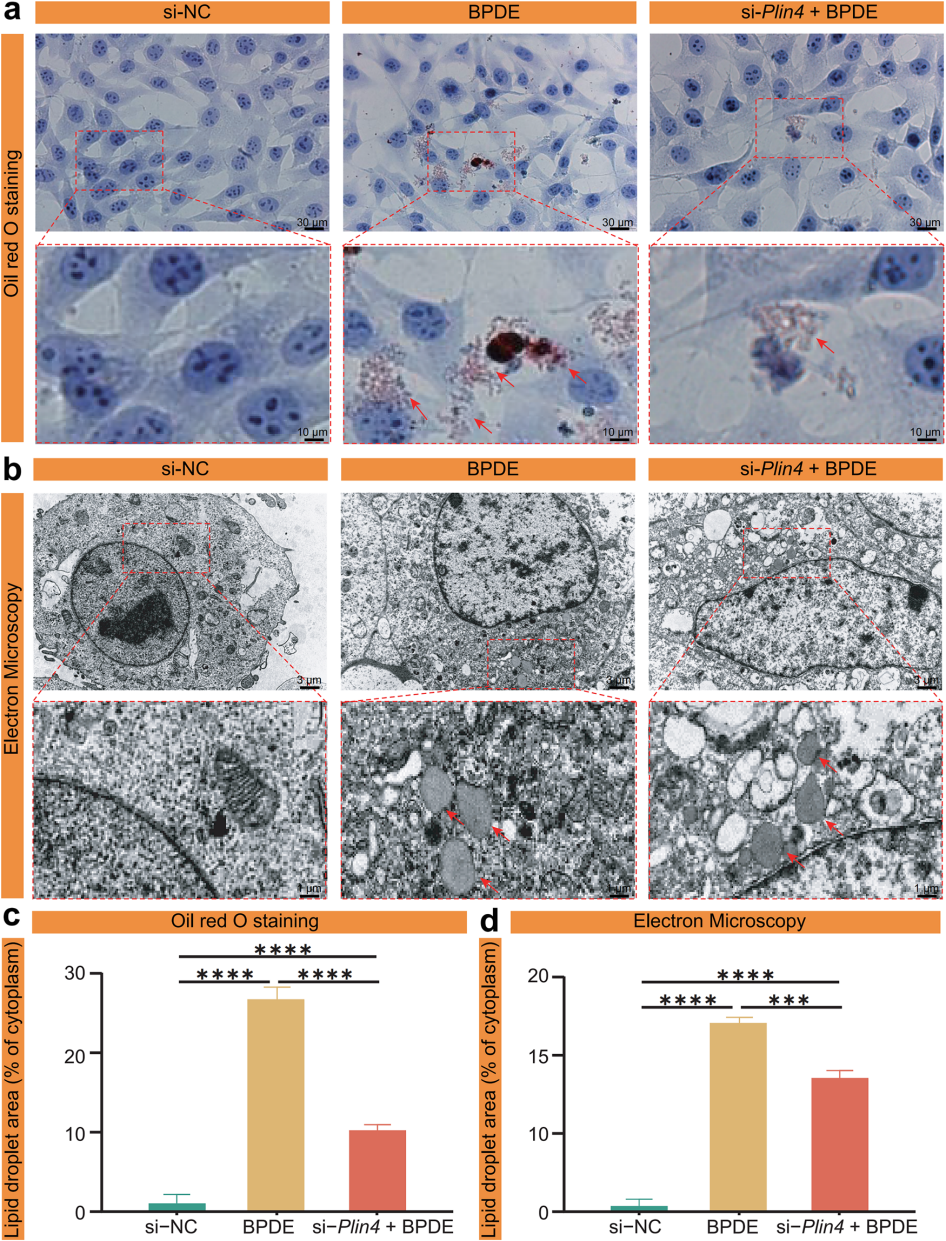
Effect of *Plin4* silencing on BPDE-induced LD accumulation and morphological changes in HT22 cells. **a** Oil Red O staining images showing LD accumulation (red arrows) in HT22 cells under different treatments: si-NC, BPDE, and si-*Plin4* + BPDE. **b** TEM images revealing ultrastructural changes in LDs (red arrows) in the same treatment groups. **c** Quantification of LD area (% of cytoplasmic area) based on Oil Red O staining images shown in (**a**). **d** Quantification of LD area (% of cytoplasmic area) based on TEM images shown in (**b**). All quantitative data correspond to the same treatment groups: si-NC, BPDE, and si-*Plin4* + BPDE. Data are presented as mean ± SEM (*n* = 6); *P < 0.05, **P < 0.01, ***P < 0.001, ****P < 0.0001.

In our study, TEM and Oil Red O staining revealed a significant increase in LDs in BPDE-treated HT22 cells, with LDs clustering around neurons and accompanied by pronounced morphological damage. Notably, *Plin4* knockdown markedly reduced LD accumulation and ameliorated cellular structural damage. These consistent findings from both ultrastructural and histological assessments suggest that *Plin4* plays a key role in BPDE-induced LD accumulation and is closely associated with neuronal damage (Fig. 5a, b).

To quantitatively evaluate the extent of LD accumulation, we performed image-based analysis using ImageJ software. For the Oil Red O staining (Fig. 5c), we measured the LD area as a percentage of the total cytoplasmic area in each cell. Similarly, for the TEM images (Fig. 5d), we quantified the proportion of LDs within the cytoplasm based on grayscale contrast and defined structural boundaries. In both analyses, at least six independent fields per group were randomly selected, and all images were processed under identical thresholding and scale parameters to ensure consistency and comparability. The quantification results showed a significant increase in LD content following BPDE treatment, whereas *Plin4* silencing significantly decreased LD accumulation. These quantitative findings further validate our qualitative observations and underscore the regulatory role of *Plin4* in BPDE-induced lipid metabolic alterations.

In conclusion, our findings indicate that BPDE promotes LD accumulation and may increase ferroptosis sensitivity through *Plin4*-regulated LD dynamics. Nevertheless, further investigation is needed to fully elucidate the mechanisms by which *Plin4*-mediated lipid metabolism contributes to ferroptosis and neuronal injury.

## Discussion

In our previous studies, we found that BPDE, the active metabolite of B[a]P, induces ferroptosis in HT22 cells and mouse primary hippocampal neurons [19, 20]. However, the specific mechanisms by which B[a]P triggers ferroptosis in neurons remain unclear. Ferroptosis is primarily characterized by reduced intracellular GSH levels, decreased antioxidant enzyme activity, and dysregulated iron homeostasis, leading to iron accumulation. Our previous study revealed that BPDE inhibits the system Xc⁻ antiporter, which consists of xCT and 4F2hc (encoded by *SLC7A11* and *SLC3A2*, respectively), thereby reducing cystine uptake, depleting intracellular GSH synthesis, inactivating GPX4, and promoting lipid peroxidation [21–23]. Furthermore, BPDE disrupts iron metabolism by elevating Fe²⁺ levels, likely through impaired iron storage or export, thereby amplifying Fenton reaction-driven oxidative damage [24, 25]. Notably, BPDE suppresses ASCL3, a transcription factor critical for neuronal antioxidant responses, exacerbating hippocampal neuronal vulnerability to ferroptosis [19, 26–28].

Recent studies suggest that BPDE-mediated system Xc⁻ inhibition may be driven by the promotion of p53 and the suppression of NRF2, both of which lead to downregulation of *SLC7A11* [21, 29]. Our group’s findings indicate that BPDE upregulates p53, a known ferroptosis enhancer that transcriptionally represses *SLC7A11*, thus decreasing cystine import and depleting intracellular GSH. Simultaneously, BPDE downregulates *NFE2L2*, a key transcription factor that normally activates *SLC7A11* expression to maintain redox homeostasis [30, 31]. This dual regulation creates a more oxidative intracellular environment, further exacerbating lipid peroxidation and ferroptosis [32].

To further dissect the role of *Plin4* in ferroptosis, we examined its impact on the expression of ferroptosis-associated genes and proteins. Our results revealed that while BPDE significantly downregulated both Slc7a11 and Gpx4 at the mRNA and protein levels, *Plin4* knockdown selectively restored the protein—but not mRNA—levels of these targets. This finding suggests that *Plin4* may regulate ferroptosis through post-transcriptional mechanisms, potentially by modulating protein translation or stability. Moreover, treatment with the ferroptosis inhibitor Fer-1 selectively rescued Slc7a11 protein expression, but had no significant effect on Gpx4 levels, indicating that Slc7a11 is more directly involved in the canonical ferroptosis pathway in this context. These results support the possibility that *Plin4* contributes to BPDE-induced ferroptosis by downregulating key ferroptosis-inhibitory proteins, especially Slc7a11, through a post-transcriptional mechanism. Thus, *Plin4* may represent a previously unrecognized modulator of ferroptosis vulnerability in neurons exposed to genotoxic stress.

Research has shown that lipid metabolism regulates ferroptosis through mechanisms such as phospholipid peroxidation and the modulation of various signaling pathways [33, 34]. Our study also focused on lipid metabolism and performed bioinformatics analysis of transcriptomic data from adult male mice exposed to acute B[a]P. We identified 1668 DEGs, and machine learning analysis revealed a gene cluster highly associated with lipid metabolism. Among these genes, *Plin4* stood out due to its significantly higher expression compared to others in the cluster. Cross-referencing with ferroptosis-related genes identified *Plin4* as a key ferroptosis-associated gene. Given its involvement in lipid metabolism, we selected *Plin4* for further investigation to better understand its role in ferroptosis.

*Plin4*, a member of the Perilipin family, is crucial for LD formation [18, 35, 36]. Increasing evidence suggests that all Perilipin proteins are involved in LD formation and regulation under various conditions [15]. Over the past two decades, lipid droplets have been characterized based on size, lipid content, and protein composition [35, 37]. LDs are highly dynamic organelles within cells, capable of storing neutral lipids such as cholesterol esters (CEs) and triacylglycerols (TGs). They have recently been recognized as essential components in stress responses across various cell types [35, 36]. In the nervous system, LDs are primarily observed in glial cells, ependymal cells, and microglia. However, under oxidative stress or lipotoxicity, LDs may also form in neurons [17, 38, 39]. The accumulation of LDs has been linked to several neurodegenerative diseases. Apart from energy homeostasis, LDs also perform various stress-related functions, including alleviating endoplasmic reticulum stress, lipotoxicity, and maintaining protein quality control. In glial cells, increased LDs have been associated with disease, and in AD models, elevated cerebral CEs and TGs correlate with LD accumulation in neural stem cell niches [17, 40]. Recent studies have highlighted the role of LDs in preventing lipotoxicity and redox imbalance (e.g., high ROS) during metabolic stress in glial cells and other cell types [14, 41].

Notably, recent studies have confirmed that *Plin4* modulates ferroptosis by inhibiting the Hippo signaling pathway. The Hippo pathway plays a crucial role in cell proliferation, apoptosis, and stress responses, and its dysregulation has been linked to ferroptosis. Specifically, inhibition of the Hippo pathway leads to the activation of Yes-associated protein (YAP), which upregulates *ACSL4*, a critical enzyme that promotes lipid peroxidation and ferroptosis. This suggests that the *Plin4*-mediated ferroptosis response may be driven, at least in part, by a Hippo-off/YAP-on/*ACSL4*-up cascade, further sensitizing cells to ferroptosis [42, 43].

In this study, we observed *Plin4* upregulation in the hippocampal tissues of B[a]P-exposed mice, mouse primary hippocampal neurons, and HT22 cells. Given previous findings that B[a]P induces ferroptosis in these models, we hypothesized that *Plin4* may contribute to this process. To test this, we knocked out *Plin4* in HT22 cells and assessed ferroptosis-related markers, including GSH content, GSH-Px activity, MDA levels, and Fe²⁺ accumulation. *Plin4* silencing attenuated ferroptosis damage and partially mitigated Erastin-induced cytotoxicity, thereby restoring cell viability [21]. These results indicate that *Plin4* is involved in mediating B[a]P-induced ferroptosis in HT22 cells.

However, one limitation of our study is the lack of assessment of Plin4 protein expression in hippocampal tissues and neuronal cells following B[a]P exposure. While *Plin4* was initially identified through transcriptomic analysis and its mRNA expression was validated by qPCR in both in vivo and in vitro models, protein-level confirmation would have further substantiated its regulatory role. Although functional experiments using gene silencing provided supporting evidence for its involvement in B[a]P-induced ferroptosis, direct quantification of Plin4 protein remains necessary to reinforce our conclusions. We appreciate this important point and will address it in future studies.

**Fig. 6:**
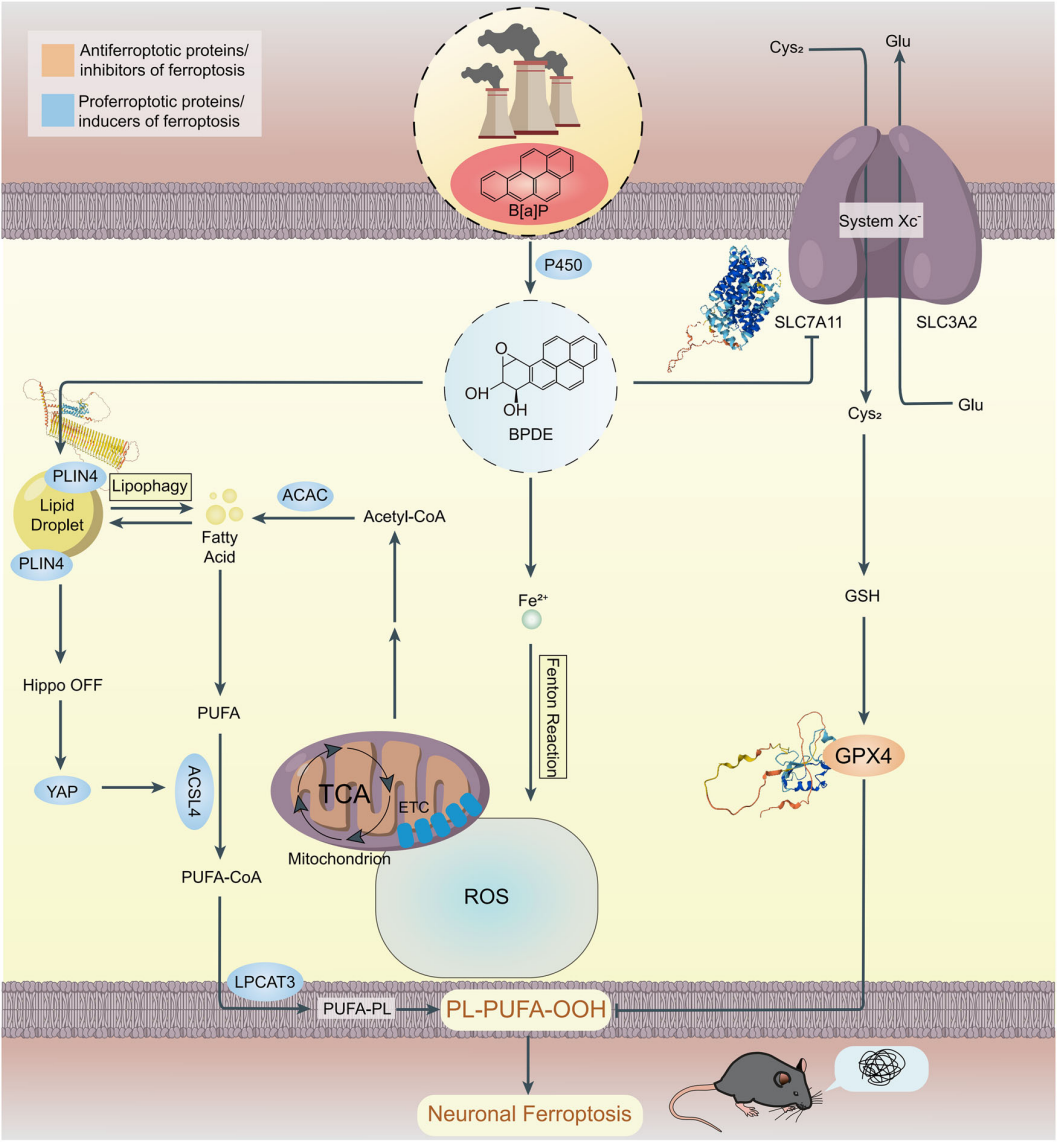
Schematic diagram of B[a]P-induced neuronal ferroptosis mechanism. This diagram illustrates how B[a]P exposure leads to the inhibition of the system Xc⁻ antiporter, depletion of GSH, iron overload, lipid peroxidation, and LD accumulation, which collectively contribute to neuronal ferroptosis.

Taken together with our previous findings, our current results support a model in which BPDE triggers neuronal ferroptosis via a triad of mechanisms: inhibition of system Xc⁻ leading to GSH depletion, Fe²⁺ overload promoting lipid peroxidation, and *Plin4*-mediated lipid droplet accumulation exacerbating oxidative stress. These interactions are illustrated in Fig. 6.

In summary, *Plin4* is identified as a critical regulator of B[a]P/BPDE-induced ferroptosis via its role in lipid metabolism. Targeting *Plin4* may disrupt this multi-hit ferroptosis pathway—addressing lipid peroxidation, iron overload, and system Xc⁻ dysfunction—and thus represents a promising therapeutic strategy for ferroptosis-associated neurodegeneration.

Despite these insights, several limitations should be acknowledged. First, the models employed—HT22 cells and acute B[a]P exposure in mice—may not fully replicate chronic or human-relevant conditions. Second, while *Plin4* was validated, other genes within the lipid metabolism network remain unexplored. Third, the upstream regulatory mechanisms and in vivo functional relevance of *Plin4* require further investigation. Future studies using chronic exposure models, conditional knockout mice, and integrated multi-omics approaches are needed to comprehensively elucidate *Plin4*’s role and therapeutic potential in neurodegenerative disease contexts.

## Materials and methods

### Transcriptome data collection and analysis

Transcriptome data were retrieved from the Gene Expression Omnibus (GEO) database (http://www.ncbi.nlm.nih.gov/geo/) using the keywords “Benzo[a]pyrene” and “mouse”. The dataset GSE75206 was selected, which includes hippocampal tissues from male mice treated with Benzo[a]pyrene (B[a]P, 70 mg/kg·bw) for three consecutive days, with tissues harvested 24 h after the final exposure. The dataset consists of eight samples (*n* = 4 control, *n* = 4 B[a]P-treated) [16]. Each sample represents a biologically independent hippocampal tissue from a separate mouse. Gene expression data were normalized before analysis, and DEGs were identified using the “Limma” package (v3.56.2) in R (v4.3.0), with thresholds set at adjusted *P*-value < 0.05 (FDR) and |log_2_FC| ≥ 3. Visualization, including heatmaps and volcano plots, was performed using the “ggplot2” package (v3.4.3).

### Gene Ontology (GO) and Kyoto Encyclopedia of Genes and Genomes (KEGG) enrichment analysis

GO and KEGG enrichment analyses were performed using the “ClusterProfiler” package (v3.18.1), “org.Hs.eg.db” (v3.11.0), and “AnnotationDbi” (v1.51.0) in R [44, 45]. Significance thresholds were set at *P* ≤ 0.05 and *q* ≤ 0.05. Results were visualized for interpretation.

### Protein-protein interaction (PPI) network

The PPI network of DEGs was constructed using the STRING database (https://cn.string-db.org/) with a combined score >0.3 [46]. Network visualization and analysis were conducted using Cytoscape software (https://cytoscape.org/), and betweenness centrality (BC) scores were calculated to identify key genes [47].

### Machine learning for candidate gene selection

The KMeans algorithm (K-means clustering) was applied to cluster genes based on expression levels and BC scores. The algorithm iteratively assigned genes to K clusters by minimizing the distance to cluster centroids until convergence [48].

### Acquisition of ferroptosis-related genes

Ferroptosis-related genes were obtained from the FerrDb database (www.zhounan.org), developed by the Affiliated Brain Hospital of Guangzhou Medical University and Sichuan University [49]. FerrDb provides curated information on ferroptosis regulators (drivers, inhibitors, markers, and unclassified factors) and modulators (inducers and inhibitors), categorized by evidence level (validated, screened, predicted, deduced).

### Venn diagram analysis

Venn diagrams were generated using the Venny 2.1 online tool (https://bioinfogp.cnb.csic.es/tools/venny/index.html) to identify overlapping genes between datasets and screen key genes for further analysis.

### Ethics statement and animal housing conditions

All animal experiments were approved by the Ethics Committee of Shanxi Medical University (Approval No. 2021053). Healthy adult male C57BL/6J mice (clean grade) were obtained from the Laboratory Animal Center of Shanxi Medical University [Certificate No. SYSK (Jin) 2015-0001]. Animals were housed in a well-ventilated facility under natural light conditions, with 40–60% relative humidity, ambient noise below 60 dB, and free access to food and water. After a one-week acclimatization period, mice were randomly assigned to two groups (*n* = 6 biologically independent animals per group): a control group receiving intraperitoneal injections of corn oil (vehicle), and a treatment group receiving 10 mg/kg body weight B[a]P dissolved in corn oil. Injections were administered once daily for 30 consecutive days to establish a subacute B[a]P exposure model. The sample size (*n* = 6 per group) was chosen based on previous literature and preliminary studies to ensure sufficient statistical power while minimizing animal use in accordance with ethical standards. Each experiment was independently repeated 6 times (biological replicates), and each biological replicate included 3 technical replicates per assay to ensure reproducibility and accuracy. Data are presented as mean ± standard error of the mean (SEM). Statistical analyses were conducted using two-sided tests with corrections for multiple comparisons where appropriate. No blinding was performed during group allocation or outcome assessment. All procedures were performed following institutional guidelines and national regulations for the care and use of laboratory animals. No animals or samples were excluded from the analysis, and no pre-established exclusion criteria were applied.

### Primary hippocampal neuron culture

Neonatal C57BL/6J mice (postnatal day 0–1) were obtained from the Experimental Animal Center of Shanxi Medical University [Certificate No. SCXK (Jin) 2019-0004; Ethics Approval No. 2021053]. After disinfection with 75% ethanol, brains were rapidly removed and placed in cold, calcium- and magnesium-free PBS. Under a stereomicroscope, meninges were carefully removed, and hippocampi were dissected out. The tissue was minced into ~1 mm³ pieces and digested with 0.25% trypsin and DNase I at 37 °C for 10 min. Digestion was terminated with DMEM containing 6% fetal bovine serum (FBS). The suspension was gently triturated, filtered, and centrifuged at 300 × *g* for 5 min. The cell pellet was resuspended in DMEM with 10% FBS and seeded onto poly-L-lysine-coated plates. After 4 h of incubation at 37 °C in 5% CO₂, the medium was replaced with serum-free Neurobasal^®^-A medium supplemented with 2% B-27. Half of the medium was changed every 3 days. Neurons were used for experiments on days in vitro (DIV) 6–7.

To simulate B[a]P exposure in vitro, cells were treated with benzo[a]pyrene diol epoxide (BPDE), the active metabolite of B[a]P, at concentrations of 0, 0.25, 0.5, or 0.75 μM for 24 h to assess its effects on cell viability and the expression of *Plin4*. All experiments were performed in 6 independent biological replicates, each with 3 technical replicates. Data are presented as mean ± SEM. Statistical analyses were performed using two-sided tests with adjustments for multiple comparisons where appropriate.

### HT22 cell culture

HT22 mouse hippocampal neuronal cells (BNCC Biological Technology Co., Ltd, BNCC358041) were cultured in Dulbecco’s Modified Eagle Medium (DMEM; Gibco, USA) supplemented with 10% fetal bovine serum (FBS; Gibco, USA) and 1% penicillin-streptomycin (Gibco, USA), maintained at 37 °C in a humidified incubator with 5% CO₂. Cells were passaged when they reached 80–85% confluence using 0.25% trypsin (Gibco, USA). For recovery, cryopreserved cells were thawed in a 37 °C water bath, centrifuged at 1200 rpm for 5 min, resuspended in complete medium, and seeded into culture flasks. For cryopreservation, cells at 80–90% confluence were harvested, centrifuged at 1000 rpm for 5 min, resuspended in freezing medium containing DMSO, and gradually frozen in a programmed freezing container.

To evaluate the cytotoxic effects of BPDE in vitro, HT22 cells were exposed to increasing concentrations of BPDE (0, 0.25, 0.5, and 0.75 μM) for 24 h. Changes in cell viability and *Plin4* expression were then assessed. For further investigation into the role of *Plin4* in BPDE-induced ferroptosis, cells were divided into experimental groups with two BPDE concentrations (0 μM and 0.75 μM). Each group was treated with either si-NC (negative control), si-*Plin4* (*Plin4* silencing), or Fer-1 (ferroptosis inhibitor) to explore the effects of *Plin4* knockdown and ferroptosis inhibition on BPDE-induced cell death and lipid peroxidation.

All in vitro experiments were performed in 6 independent biological replicates, and each biological replicate included 3 technical replicates. Data are expressed as mean ± SEM. Statistical analyses were conducted using two-sided tests, with corrections for multiple comparisons where applicable. Statistical significance was considered at *P* < 0.05. HT22 cells were authenticated by the supplier and tested for mycoplasma contamination prior to use.

### Morris water maze (MWM) test

The MWM apparatus (purchased from the Department of Environmental Toxicology, Shanxi Medical University) consisted of a 150 cm diameter, 50 cm high pool with an overhead camera tracking system. The pool was divided into four quadrants (SW, NW, SE, NE), with water maintained at 22 ± 1 °C and a platform submerged 1 cm below the surface. After 1-h dark-room acclimation, mice underwent four daily training trials for 5 days, starting from different quadrants, with 60 seconds to locate the hidden platform. Escape latency was recorded, and mice remained on the platform for 15 s; failures were guided to the platform for a 20 s stay. On day 6, a spatial probe test was conducted by removing the platform and allowing mice to swim freely for 60 s. The swimming path and time spent in the target quadrant (SW) were recorded.

### Y-Maze test

The Y-Maze apparatus (black acrylic, purchased from the Department of Environmental Toxicology, Shanxi Medical University) consisted of three arms at 120° angles: starting arm, other arm, and novel arm. In the first phase, mice explored the starting and other arms for 5 min, with the novel arm blocked. After a 1 h rest, they were reintroduced and allowed 5 min to explore all three arms. Between trials, the maze was cleaned with 70% ethanol. A video tracking system recorded behavior, and the number of entries and latency to enter the novel arm were analyzed.

### Quantitative real-time PCR (qPCR)

Total RNA was extracted using RNA isolator Total RNA Extraction Reagent (Vazyme, R701-01, Nanjing), and cDNA was synthesized with HiScript II Q Select RT SuperMix for qPCR (+gDNA wiper) (Vazyme, R222-01, Nanjing) following the manufacturer’s instructions. qPCR was performed using PowerUP SYBR Green Master Mix (Thermo Fisher, USA) on an ABI StepOne Plus system (Applied Biosystems, USA). The thermal cycling conditions were as follows: initial denaturation at 95 °C for 5 min, followed by 40 cycles of 95 °C for 10 s and 60 °C for 30 s, with fluorescence signal acquisition during each extension phase. Melting curve analysis was conducted to verify specificity. The primer sequences were as follows:

- *Plin4*
  Forward: 5′-GTGTCCACCAACTCACAGATG-3′
  Reverse: 5′-GGACCATTCCTTTTGCAGCAT-3′
- *Slc7a11*
  Forward: 5′-GGCACCGTCATCGGATCAG-3′
  Reverse: 5′-GGACCAAAGACCTCCAGAATG-3′
- *Gpx4*
  Forward: 5′-TGTGCATCCCGCGATGATT-3′
  Reverse: 5′-CCCTGTACTTATCCAGGCAGA-3′
- *Ptgs2*
  Forward: 5′-TTCCAATCCATGTCAAAACCGT-3′
  Reverse: 5′-AGTCCGGGTACAGTCACACTT-3′
- *Gapdh (endogenous control)*

   Forward: 5′-AGGTCGGTGTGAACGGATTTG-3′

   Reverse: 5′-TGTAGACCATGTAGTTGAGGTCA-3′

Relative gene expression levels were calculated using the 2^−ΔΔCt^ method.

### Cell viability assay

HT22 cells were seeded into 96-well plates at a density of 1 × 10⁵/mL to 1 × 10⁶/mL. Upon reaching appropriate confluence, cells were treated according to experimental groups. After 24 h, 10 μL of CCK-8 (Cell Counting Kit-8) solution was added to each well and incubated for 2 h at 37 °C in a 5% CO₂ incubator. Absorbance at 450 nm was measured using a microplate reader. Cell viability was calculated using the formula: Cell viability = [(Experimental group-Blank control)/(Control group-Blank control)] × 100%. Three replicate wells were used per group, with a blank control well.

### Establishment of the cell gene silencing model for *Plin4*

*Plin4* gene silencing was performed using siRNA and transfection reagents from GenePharma (Shanghai). Before transfection, 500 μL of growth medium was added to each well, and 1.5–3.5 × 10⁴ cells were seeded to reach 30–50% confluence 18–24 h prior. The siRNA-mate transfection reagent was mixed with 10 pmol (140 ng) of siRNA targeting *Plin4* in 100 μL DMEM medium and vortexed. After a 10 min incubation at room temperature, 100 μL of the transfection mixture was added to each well, resulting in a final siRNA concentration of 16.7 nM. Cells were incubated at 37 °C, and *Plin4* silencing efficiency was assessed by qPCR at 24–72 h post-transfection.

### Cell morphology and ultrastructure

Ultrastructural analysis of cells was performed using TEM. After treatment, cells were centrifuged at 1200 rpm for 5 min, fixed in 2% glutaraldehyde for 2 h, and washed three times with PBS. The cells were dehydrated with graded acetone solutions, followed by infiltration and embedding. Thin sections were stained with 3% uranyl acetate and lead citrate, and the ultrastructural features were observed and imaged using a TEM.

### Western blot

Proteins were separated using SDS-PAGE with gels corresponding to the target molecular weights, loading 10–20 μg protein per lane. The electrophoresis was carried out at 80 V for 30 min and then at 120 V until dye migration. Proteins were transferred to NC membranes using a wet transfer system (350 mA, 90 min) in Tris-glycine buffer. Membranes were blocked with 10% skim milk in TBST for 1 h and incubated overnight at 4 °C with the following primary antibodies: anti-SLC7A11 (ab307601, Abcam, 1:1000), anti-GPX4 (ab125066, Abcam, 1:2000), and anti-GAPDH (10494, Proteintech, 1:10 000). After TBST washes, membranes were incubated with HRP-conjugated secondary antibody (anti-rabbit IgG, AS014, Abclonal, 1:10,000) for 1 h at room temperature. Protein signals were visualized using ECL substrate (1:1 reagent A:B mixture) and quantified via ImageJ by normalizing target band intensities to loading controls. Full and uncropped western blots are shown in Supplementary Material.

### Glutathione (GSH) content detection

The GSH content in HT22 cells and primary cells was measured using a colorimetric method. Reduced GSH reacts with 5,5′-dithiobis (2-nitrobenzoic acid) (DTNB) to form a yellow compound, quantified at 405 nm. After treatment, cells were lysed with RIPA buffer, and the supernatant was collected for analysis. Protein concentration and GSH content were measured according to the manufacturer’s protocol, and absorbance was read at 405 nm using a microplate reader.

### Glutathione peroxidase (GSH-Px) activity detection

The GSH-Px activity in cells was measured using an enzyme-catalyzed colorimetric method. Reduced GSH reacts with DTNB to form a yellow compound, quantified at 412 nm. Cells were lysed as described, and GSH-Px activity was assessed using the manufacturer’s protocol. Absorbance was measured at 412 nm with a microplate reader.

### Measurement of cellular Lipid ROS

HT22 cells and primary cells were seeded in 96-well plates and treated as per experimental groups. After 24 h, cells were washed with PBS and incubated with 8 μM C11-BODIPY (a fluorescent probe for lipid oxidation) at 37 °C for 30 min. Fluorescence signals for oxidized and non-oxidized lipids were measured using a multimode plate reader (non-oxidized: excitation/emission 540 nm/620 nm; oxidized: excitation/emission 428 nm/528 nm). The lipid ROS level was calculated by the ratio of oxidized to total fluorescence.

### Malondialdehyde (MDA) content detection

MDA content in cells was measured using the thiobarbituric acid method. Cells were lysed with RIPA buffer, and the supernatant was collected for analysis. After adding the reagent mixture and sonication, the sample was incubated in a 95 °C water bath for 40 min, followed by cooling and centrifugation. Absorbance was measured at 532 nm using a microplate reader, and MDA content was calculated using the appropriate formula.

### FerroOrange staining

HT22 cells were seeded at 100,000 cells/mL in confocal culture dishes and cultured overnight at 37 °C with 5% CO₂. After discarding the medium and washing with serum-free medium, cells were treated for 24 h as per the experimental groups. The cells were then stained with 1 μM FerroOrange (a fluorescent dye for detecting iron) for 30 min. Staining was observed and analyzed using a confocal microscope.

### Ferrous Iron (Fe^2+^) content detection

HT22 cells were seeded in 96-well plates and treated as per the experimental design. After washing with serum-free medium, cells were incubated with 1 μM FerroOrange for 30 min at 37 °C. Fe²⁺ content was measured using a fluorescence microscope, with excitation at 542 nm and emission at 575 nm. Fluorescence intensity was quantified to assess intracellular Fe²⁺ levels.

### Oil Red O staining

HT22 cells were cultured and treated as per the experimental design. After removing the culture medium and washing with PBS, cells were fixed with Oil Red O fixative for 20–30 min, followed by washing with distilled water. The cells were immersed in 60% isopropanol for 20–30 s and then stained with Oil Red O solution for 10–20 min. Mayer’s hematoxylin was applied for 1–2 min, and the cells were washed with Oil Red O buffer. Staining was observed under a microscope.

### LD quantification

LD accumulation was quantitatively analyzed using ImageJ (National Institutes of Health, USA). For Oil Red O staining, images were captured under a bright-field microscope at the same magnification and exposure settings across all groups. The cytoplasmic region of each cell was manually outlined as the region of interest (ROI), and the total LD area was quantified by converting the image to 8-bit grayscale, followed by threshold adjustment to isolate positively stained regions. The LD area was then measured and expressed as a percentage of the total cytoplasmic area for each cell. At least six randomly selected fields per group were analyzed.

For TEM images, LDs were identified based on their electron-lucent appearance and spherical morphology. TEM images were converted to grayscale and analyzed using a similar thresholding approach in ImageJ. LD areas were measured and normalized to the total cytoplasmic area within each image. For each experimental group, six biological replicates (*n* = 6) were analyzed, and three independent regions were selected from each replicate for quantification. Quantitative values from all selected regions were averaged to calculate the LD content for each group. All image processing and analysis were performed using identical parameters across all groups to ensure consistency. Data are presented as mean ± SEM.

### Quantification and statistical analysis

All data were analyzed using GraphPad Prism (Version 9.0, GraphPad Software, USA). Results are presented as mean ± SEM, unless otherwise specified. Each experiment was independently repeated six times (biological replicates), with three technical replicates per biological sample. For comparisons between two groups, two-sided unpaired Student’s t-tests were performed. For comparisons among multiple groups, one-way analysis of variance was used, followed by Tukey's post hoc test for multiple comparisons. Homogeneity of variances was confirmed prior to performing parametric tests. A *P*-value < 0.05 was considered statistically significant. Exact sample sizes (n), statistical tests used, and significance levels are detailed in the corresponding figure legends.

The sample sizes were chosen based on prior published studies and preliminary experiments conducted in our laboratory to ensure adequate statistical power to detect biologically relevant differences. Although formal power calculations were not performed, the selected group sizes (*n* = 6 biological replicates) are consistent with similar studies in the field. This, combined with three technical replicates per biological replicate and independent repetition of experiments, provides confidence in the reproducibility and robustness of the findings.

### Reporting summary

Further information on research design is available in the Nature Portfolio Reporting Summary linked to this article

## Supplementary information

Full and uncropped western blots

## Data Availability

The dataset analyzed in this study, GSE75206, was retrieved from the Gene Expression Omnibus (GEO) database. Additional data supporting the conclusions of this work are available from the corresponding author upon reasonable request.

## Code availability

R codes used to analyze data and generate figures are available at Github (https://github.com/Sun0shy/BPDE_Ferroptosis).
